# The Effects of Temperature and Humidity on the Microstructure of Sulfonated Syndiotactic–polystyrene Ionic Membranes

**DOI:** 10.3390/membranes10080187

**Published:** 2020-08-14

**Authors:** Maria-Maddalena Schiavone, David Hermann Lamparelli, Yue Zhao, Fengfeng Zhu, Zsolt Revay, Aurel Radulescu

**Affiliations:** 1 Forschungszentrum Jülich GmbH, Jülich Centre for Neutron Science (JCNS) at Heinz Maier-Leibnitz Zentrum (MLZ), 85747 Garching, Germany; schiavonemariamaddalena@yahoo.it (M.-M.S.); f.zhu@fz-juelich.de (F.Z.); 2 Dipartimento di Chimica e Biologia “Adolfo Zambelli”, Università di Salerno, I-84084 Fisciano, Italy; dlamparelli@unisa.it; 3 Department of Advanced Functional Materials Research, Takasaki Advanced Radiation Research Institute, National Institutes for Quantum and Radiological Science and Technology (QST), Watanuki-machi 1233, Takasaki 370-1292, Japan; zhao.yue@qst.go.jp; 4 Technische Universität Müchen, Forschungsneutronenquelle Heinz Maier-Leibnitz FRM II, Heinz Maier-Leibnitz Zentrum (MLZ), 85747 Garching, Germany; Zsolt.Revay@frm2.tum.de

**Keywords:** proton exchange membranes, semi-crystalline polymers, small-angle neutron scattering

## Abstract

Polymeric membranes based on the semi-crystalline syndiotactic–polystyrene (sPS) become hydrophilic, and therefore conductive, following the functionalization of the amorphous phase by the solid-state sulfonation procedure. Because the crystallinity of the material, and thus the mechanical strength of the membranes, is maintained and the resistance to oxidation decomposition can be improved by doping the membranes with fullerenes, the sPS becomes attractive for proton-exchange membranes fuel cells (PEMFC) and energy storage applications. In the current work we report the micro-structural characterization by small-angle neutron scattering (SANS) method of sulfonated sPS films and sPS–fullerene composite membranes at different temperatures between 20 °C and 80 °C, under the relative humidity (RH) level from 10% to 70%. Complementary characterization of membranes was carried out by FTIR, UV-Vis spectroscopy and prompt–γ neutron activation analysis in terms of composition, following the specific preparation and functionalization procedure, and by XRD with respect to crystallinity. The hydrated ionic clusters are formed in the hydrated membrane and shrink slightly with the increasing temperature, which leads to a slight desorption of water at high temperatures. However, it seems that the conductive properties of the membranes do not deteriorate with the increasing temperature and that all membranes equilibrated in liquid water show an increased conductivity at 80 °C compared to the room temperature. The presence of fullerenes in the composite membrane induces a tremendous increase in the conductivity at high temperatures compared to fullerenes-free membranes. Apparently, the observed effects may be related to the formation of additional hydrated pathways in the composite membrane in conjunction with changes in the dynamics of water and polymer.

## 1. Introduction

Polymer electrolyte materials used as proton exchange membranes (PEM) for fuel cells applications (PEMFC) are characterized by a nanoscale phase separation into hydrophilic domains and hydrophobic regions, which is a combination that enables a high, water-mediated, proton conductivity and provides a good chemical and mechanical stability, and thus membrane durability [1,2,3]. Although it is characterized by excellent conductive properties, the Nafion, which was established as a benchmark for such applications [4], presents also some drawbacks such as high cost, lack of safety, and the requirement of supporting equipment during manufacturing and use [5]. Furthermore, it shows limitations under operating conditions at high temperature (> 80 °C) and low relative humidity (RH), when a decrease in conductivity appears due to dehydration of the membrane at the anode side [5]. Moreover, free radicals such as hydroxyl and hydroperoxyl are produced during the operation of the PEMFC as a result of the reaction of hydrogen and oxygen on the electrodes or the decomposition of hydrogen peroxide with metal contaminants in the membrane. These radicals initiate processes of chemical degradation that affect the durability and the lifetime of the PEM [6,7]. Therefore, improvements in the Nafion properties by incorporating inorganic fillers (such as titania, zirconia, and silica) or carbon-based nanomaterials (carbon nanotubes, fullerenes, etc.) into the membranes [7,8,9,10,11,12,13,14] and alternative low-cost materials that present similar conductive and chemo-mechanical properties, such as the Nafion membranes [14,15,16,17,18,19,20,21,22,23,24,25], are continuously searched for. Given the recent developments, which enable a controlled sulfonation of only the amorphous phase [26], preserving thus the crystallinity of the material, and an improved resistance to oxidation decomposition when fullerenes are added [27], the sulfonated syndiotactic polystyrene (s–sPS) is a good potential candidate for some PEMFC applications, as it presents a high proton conductivity comparable to Nafion [28], high chemical and thermomechanical stability, and a low cost. The preparation of a s–sPS membrane should start from the δ-form (clathrate with guest molecules), which enables the functionalization of the phenyl groups in the amorphous region and can be subsequently transformed into the thermodynamically stable β-form by high temperature annealing procedures. The s–sPS membranes present a strong dependence of the proton conductivity on sulfonation level, temperature and hydration conditions [29,30]. 

The proton conduction in PEMs depends on water and is governed by the water behavior at different length scales: at the molecular scale: dissociation of protons and formation of the ion-pair with water, at the nanoscale: the transport through the hydrated domains, at the mesoscale: the long-range mobility within the water network [4]. Therefore, in order to understand the transport properties in different conditions, one should first of all learn about the morphology of hydrated domains at different length scales as a function of hydration level and temperature. The microstructure of PEM materials and the elucidation of the conductive paths at the microscopic level are highly debated topics. In previous works [31,32] we reported a detailed microstructural characterization of s–sPS membranes with δ–clathrate co–crystalline form, which was carried out by extended Q-range small-angle neutron scattering, SANS, where Q = 4 π *λ*^−1^ sin(θ/2) is the modulus of the scattering vector Q, with *λ* the incident neutron wavelength and θ the scattering angle. Membranes with different sulfonation degrees were investigated in dry and hydrated states at room temperature. A humidity chamber (Anton Paar, Graz, Austria) was used to control the relative humidity (RH) degree between 5% and 95%. The use of uniaxially deformed film samples and neutron contrast variation allowed for the identification and characterization of different structural levels with sizes between nm and μm, which form and evolve with the variation of the hydration level. The neutron scattering length density (SLD) of the crystalline regions was varied using different toluene isotopologues incorporated as guest molecules into the sPS lattice [33], while the variation of the scattering properties of the hydrated amorphous regions was achieved using different H_2_O/D_2_O mixtures. Deuterated s–sPS films were used in the investigation to obtain a low incoherent background. According to our qualitative and quantitative analysis of the SANS data in terms of structural models, the hydration water is taken-up at low RH in clusters formed around the sulfonic groups. The clusters in the bulk amorphous region grow in size with increasing hydration level, favored by the increased flexibility of the sPS chains in these domains. At very high hydration level, towards RH = 100%, the clusters become interconnected one with another, which gives rise to the formation of cylindrical channels morphology. Observations made by cryo-TEM on fully hydrated films support the SANS conclusions [31]. Moreover, the neutron contrast variation measurements revealed that the hydrated ionic clusters promoting the conductivity of the membranes include the segments of the sPS chains that are affected by sulfonation, hence the phenyl-groups too. The extended Q-range SANS data have also shown that the crystallinity, and hence the robustness of the membrane, is preserved during drying-hydration processes: the 010 crystalline reflection, which is indicative of the crystalline δ–form, was always observed in the scattering pattern, no matter which contrast or hydration conditions were used, while the scattering pattern that is characteristic of a dry membrane was always recovered after exposing the membrane to different hydration procedures. 

In this paper, we discuss the temperature effect on the microstructure of the s–sPS membranes at different hydration levels, well below the full hydration state, based on results of a SANS investigation done on cast and uniaxially deformed s–sPS semi-crystalline films. The films consist of the δ–co–crystalline phase of sPS with deuterated toluene and an amorphous phase in which C60 fullerenes have been incorporated. A detailed qualitative analysis was carried out on the cast films, which were studied at different temperatures between 20 °C and 80 °C, with the variation of the hydration level at every temperature between RH = 10% and RH = 80%. Quantitative structural and water content information were extracted from the analysis of the scattering data from the uniaxially deformed films in terms of structural models, which were also used before [31,32]. The use of uniaxially deformed films enabled the separation of the scattering contribution from different components of the hydrated morphologies on the two-dimensional SANS detector while covering different angular scattering ranges, as detailed in our earlier works. Usually, for a thorough characterization of complex multiphase systems such as polymeric membranes used in energy and biomedical applications [32,34], a combination of experimental techniques is necessary and enables the exploration of the microstructure, morphology and composition properties in various chemical and thermodynamic conditions and their relationship with macroscopic properties of interest for particular applications. Complementary information about the composition and crystallinity of the sPS-based membranes were delivered by Fourier-transform infrared spectroscopy (FTIR), X-ray diffraction (XRD) and prompt-gamma neutron activation analysis (PGAA). Finally, the conductivity of membranes was measured on the cast films equilibrated in liquid water at different temperatures. First insights were obtained about the structural changes induced by the variation of temperature and hydration in the membrane morphology. On the other hand, the incorporation of fullerenes in the composite membrane induces a tremendous increase in membrane conductivity at high temperatures compared to fullerenes-free membranes. No evidence to explain this effect was obtained from the micro-structural analysis at this level. Apparently, this effect may be related to the formation of additional hydrated pathways in the composite membrane in conjunction with changes in the dynamics of water and polymer. 

## 2. Materials and Methods 

The preparation and subsequent treatment—clathration, sulfonation, and guest-exchange in the crystalline region of uni-axially-oriented deuterated syndiotactic polystyrene films were described elsewhere [31]. For the SANS experiment performed in this study, the exchange of the guest molecules in the polymer clathrate form from d–chloroform, which was loaded during the sulfonation procedure, to d-toluene, was achieved by dipping the films for 1 day in the new solvent, followed by drying at 40 °C under vacuum for a couple of hours.

To prepare the sPS, C60 composite membranes required amounts of newly synthesized deuterated sPS [31], and commercially achieved C60 fullerenes (Merck, Darmstadt, Germany) and h–toluene (ARMAR Chemicals, Döttingen, Switzerland) were taken in hermetically sealed tubes and heated at 170 °C for several minutes, until the solution became homogeneous. The resultant solutions were cast uniformly on quartz substrates to form sPS–C60 composite membranes. Membranes were prepared with a different C60 content in the initial solution, ranging from 0.05 wt% to 1 wt%. The sPS–C60 composite membranes were subsequently functionalized by soaking the films in acyl sulfate solution in d–chloroform (ARMAR Chemicals, Döttingen, Switzerland) at 40 °C. The sulfonation agent was prepared according to the procedure described in [26,28]. Afterwards, the samples were removed from the solution and quickly dipped in acetone for a few minutes to remove traces of impurities (due to the possible remnants of the sulfonation procedures) and dried under the fume-hood for 24 h. The guest exchange from d–chloroform, which replaced the h–toluene, the original guest into the sPS cavities, during the sulfonation procedure, to d–toluene was carried out, to simplify the neutron contrast conditions for the SANS experiments, as discussed in [32].

As proceeded in our previous studies [31,32], the membranes were further on analyzed by FTIR, UV-Vis spectroscopy and prompt–γ neutron activation analysis in terms of composition following the specific preparation and functionalization procedure, and by XRD with respect to crystallinity.

The degree of sulfonation, expressed as S atoms/styrene units × 100 mol% and further indicated as *S*, was checked at the neutron prompt-gamma activation analysis (PGAA) instrument at the Heinz Maier-Leibnitz Zentrum (MLZ, Garching, Germany). Full descriptions of the experimental method and data interpretation can be found in [35]. 

Qualitative analysis of the sulfonation was checked by FTIR spectroscopy using a JASCO VIR-200 spectrometer (JASCO Deutschland, Pfungstadt, Germany) in a wavenumber range of 400–4000 cm^−1^. The film samples were placed in special holders equipped with ZnSe windows. 

To check the incorporation of C60 fullerenes in the membranes, UV-Vis analysis was carried out at a Cary 100 SCAN UV-Vis Varian spectrometer (Agilent, Santa Clara, CA, USA) with the film samples placed in a specific holder equipped with quartz windows. The spectra were collected in the range 200–800 nm at a resolution of 100 nm/min. 

WAXD analysis of films was done in the range of 2θ between 5° and 35° by means of an X-ray powder diffractometer Brucker 2nd Gen-D2 Phaser (Cu-source) (Brucker, Karlsruhe, Germany). The degree of crystallinity was determined as 100 A_c_/(A_c_ + A_a_), where A_c_ and A_a_ are the areas determined by resolving the diffraction pattern according to [36] and can be considered proportional to the crystalline and amorphous fractions of the polymer. 

The water uptake capacity of the membranes was determined as following the preparation procedure described in [31]. The water uptake was calculated as the percentage increase in mass over the “dry” weight:

(1) $$W_{\mathrm{uptake}}\left(w\%\right)\;=\;\left[\left(W_{\mathrm{wet}}\;-\;W_{\mathrm{dry}}\right)/W_{\mathrm{dry}}\right]\;\times \;100\%$$

where Wwet and Wdry are the wet and dry weight of the membrane, respectively. 

The conductivity of the membranes was measured in the plane direction at 100 kHz using four-point probe alternating current electrochemical impedance spectroscopy (EIS) with an electrode system connected to an LCR meter (HIOKI 3522 LCR HiTESTER, Nagano, Japan). Membranes were placed between two platinum electrodes and equilibrated in liquid water. The membrane’s conductivity was measured at room temperature and 80 °C. The conductivity *σ* (mS/cm) was calculated from the obtained resistance *R* (Ω) according to the following equation.

(2) $$\sigma \;\left(\mathrm{mS}/\mathrm{cm}\right)\;=\;L/\left(S\;\times \;R\right)\;\times 10^{3}$$

where *L* (cm) is the distance between two electrodes, and *S* (cm^2^) is the cross-sectional area of the membrane obtained by multiplying the membrane thickness by the membrane width.

The SANS measurements were carried out at the KWS-2 high intensity/extended-Q range pinhole SANS diffractometer (Forschungszentrum Jülich GmbH, Jülich, Germany) of JCNS at MLZ [37]. A Q-range between 0.003 and 0.6 Å^−1^ was covered by using three sample-to-detector distances, L_D_ = 2 m, 4 m and 20 m, and a neutron wavelength *λ* = 5 Å. The film samples were exposed to in-situ controlled hydration and temperature variation by using a humidity chamber (Anton Paar, Graz, Austria). The temperature on the sample was varied between 20 °C and 80 °C, while the relative humidity at a fixed temperature value was varied within the range RH = 10% to 70%. The data treatment and interpretation according to structural models was done according to models and calculations, which are described in details in [32].

Finally, a uniaxially deformed s–sPS membrane loaded with C60 fullerenes, which was characterized by SANS in a previous study [32], was exposed to Fenton’s test conditions [4,14,27] for 1 h at 60 °C and then briefly re-investigated with SANS (at KWS-2 diffractometer, Jülich, Germany) to get a first insight about the effect of the peroxyl/hydroxyl radicals, formed from the decomposition of H_2_O_2_ in an aqueous solution catalyzed by Fe^2+^/Fe^3+^, on the crystalline-amorphous morphology of the membrane. The Fenton’s test represents a useful method for a preliminary assessment of the oxidative stability of PEMs. Our interest in the current work was limited only to the observation of possible changes in the scattering patterns, as a consequence of an eventual degradation of the membrane after the application of the test.

## 3. Results

### 3.1. Composition Characterization

Two sPS-based membranes were characterized prior to their investigation by SANS: sample A —a uniaxially deformed s–sPS film, and sample B—a composite sPS–C60 undeformed sulfonated film, which was prepared at a 0.5 wt% C60 content from the initial common solution with sPS in h–toluene. The membranes are based on deuterated sPS and the clathrate form in the investigated samples consisted of δ–co–crystals of sPS with d–toluene. The PGAA analysis [30] delivered a sulfonation degree of about S = 45% and 41% for the samples A and B, respectively. 

**Figure 1:**
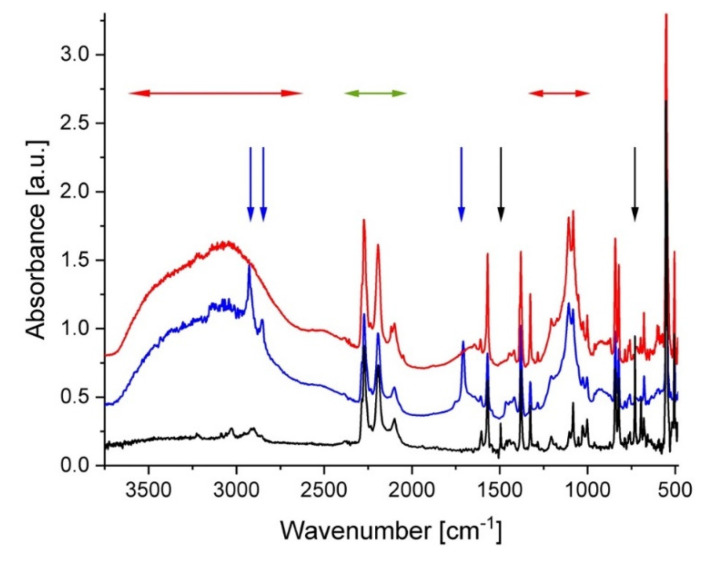
FTIR spectra from film sample B after different steps of the preparation and treatment procedure: black—after casting; blue—after functionalization (sulfonation); red—after dipping in d–toluene (to provide the exchange of guest molecules in the clathrate form). The arrows indicate the regions of interest for IR bands characteristic of different molecular groups, as discussed in the text. The spectra are shifted vertically for clarity.

The FTIR spectra from a membrane prepared in a similar way to sample A can be found in [31]. The FTIR observations done at different stages of the preparation procedure indicated the successful treatment and functionalization of the membranes of this type. The FTIR spectra from sample B are shown in Figure 1 as collected at different stages of preparation. The low spectrum (black line) was acquired from the sPS–C60 composite membrane after casting from the common solution in h–toluene. Typical spectral features of δ–form of deuterated sPS [38] can be observed, such as the ring stretching and bending modes and backbone C–D and C–D_2_ stretching in the region of 2300 to 2150 cm^−1^ (the range indicated by the green horizontal arrows) and the C=C stretching at around 1570 cm^−1^ [39]; while at short wavenumbers, between 500 and 550 cm^−1^, the bands characteristics of the chain conformation of deuterated sPS in the δ–crystalline form are displayed. These features appear in all further spectra regardless of treatment procedure. As mentioned in our previous work [32], due to the multitude of characteristic bands of sPS, it is very difficult to observe the IR bands of fullerenes. Experimental FTIR characterization of fullerenes in bulk or functionalized polymers can be found in [40] for C60 or [41] for C70, while theoretical calculations were done in [42]. The bands observed at around 1500 cm^−1^ (C–C stretching in the aromatic ring) and around 740 cm^−1^ (out-of-plane C–H bending) that are indicated by the black vertical arrows can be ascribed to vibrations characteristic of the h–toluene molecules, which are trapped in the cavities between the sPS helices (δ–clathrate form). The middle spectrum in Figure 1 (blue) was collected from the same sample after its sulfonation and washing with acetone. The two very broad features observed in the wavenumber ranges of 2500–3700 cm^−1^ and 1000–1250 cm^−1^ (marked with the red horizontal arrows) are indicative of the sulfonation of the sample. On the other hand, during the sulfonation procedure the h–toluene was replaced by the d–chloroform in the clathrate form. Accordingly, the IR band characteristics of h–toluene are not visible anymore in the spectrum, while those from d–chloroform cannot be easily distinguished from those of the deuterated sPS. Additional sharp spectral features due to h–acetone were observed (indicated by the blue vertical arrows). The IR bands in the region 3000 to 2800 cm^−1^ can be ascribed to the C–H stretching modes, while the strong feature at around 1700 cm^−1^ is due the C=O stretching.

The washed membrane, which was dipped in d–toluene subsequently to its sulfonation, delivered the IR spectrum shown in red in Figure 1. While the spectral features, which are indicative of the membrane sulfonation, are clearly visible, besides those from the deuterated sPS, the bands from the acetone or other washing agents are not present anymore. The spectral features from d–toluene are not distinguishable from the deuterated sPS bands. 

**Figure 2:**
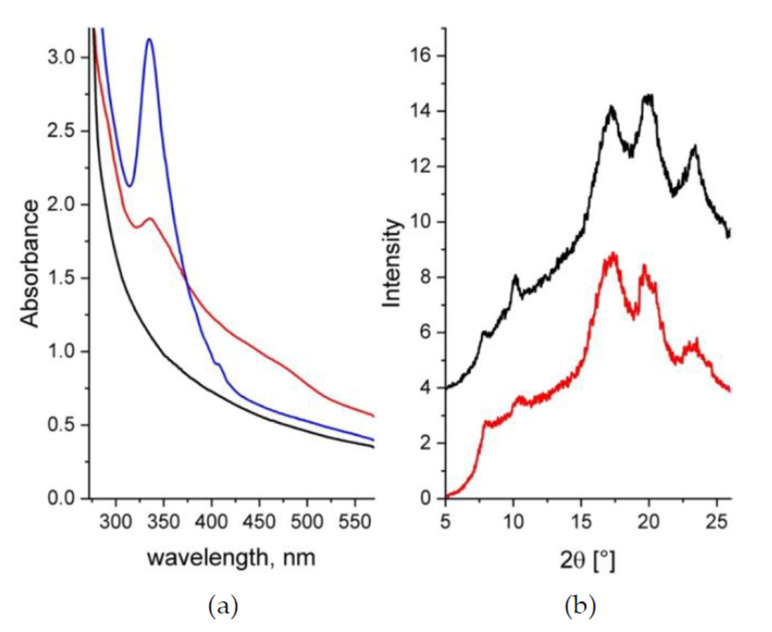
UV-Vis spectra (**a**) and XRD patterns (**b**) from samples A and B. In both panels the black (sample A) and the red (sample B) data are from samples in the final stage of treatment (sulfonated and containing d–toluene in the clathrate form), while the blue curve in the Figure 2a is from the sample B as cast (before functionalization). The XRD patterns are shifted vertically for clarity.

The UV-Vis absorption spectra of the samples A and B are shown in Figure 2a. The UV-Vis analysis was done on sample B prior (blue curve) and after (red curve) its sulfonation. The characteristic absorption features of s–sPS (sample A) occur below 300 nm while above this value the absorbance falls quickly off. Pure C60 exhibits two maxima at 335 and 408 nm [43]. These features are well displayed by the spectrum from the sPS–C60 composite membrane after casting (blue curve). This is indicative of the incorporation of fullerenes in the membrane. Although still indicating a strong absorption above 300 nm, the spectrum from the same membrane after its functionalization (red curve) presents less pronounced and rather broad features compared to its state before sulfonation. Thus, the band at 335 nm, which is known to be affected by the environment of fullerenes [44,45], is broader and less intense, while the small peak at 408 nm completely disappears, and a new broad shoulder-like absorption between 400 and 500 nm appears. The excess absorption above 400 nm seems to be due to agglomeration of the C60 fullerenes. As reported in [44], there is a propensity shown by fullerenes to aggregation that depends on sample preparation and treatment procedures. It seems that the effect observed in the UV-Vis spectra from functionalized sPS/C60 composite membranes, which looks similar to those observed from fullerenes-doped s–sPS membranes [32], is a consequence of the sulfonation of the membrane. However, a clear understanding of this effect requires further investigation.

The WAXD spectra from the samples A and B in their final treatment stage, after functionalization and exchange of the guest molecules in the clathrate form to d–toluene, are presented in Figure 2b. The pair of peaks at around 8° and 10.5° in 2θ is indicative of the formation of the crystalline δ–form of the sPS clathrates with guest molecules [46,47]. The presence of these peaks in the patterns collected from both samples indicates that the sPS crystalline habit is preserved, regardless of treatment. The positions of the diffraction peaks are the same in all WAXD patterns, which indicates that the addition of fullerenes does not change the parameters of the polymer crystalline lattice. Evidences about the incorporation of C60 within the crystalline lattice of sPS were reported in [47] following the analysis of the XRD patterns from sPS–C60 composite samples with higher C60 content than our membranes. The additional peaks that were observed besides those from the δ–form of the sPS were attributed to an FCC arrangement of C60. Although we did not observe any additional peaks in the XRD patterns from our film samples, incorporation of some of the C60 within the polymer crystalline lattice cannot be completely ruled out. Although in this case fullerenes may compete with the d–toluene molecules in occupying the cavities between the sPS helices in the clathrate form, however, because of the rather similar neutron scattering length density (SLD) of C60 and d–toluene [32], no change in the neutron scattering properties of the crystalline regions is expected for the SANS experiments. Finally, from the analysis of the WAXD patterns in terms of the A_c_ (peaks) and A_a_ (background) areas [36], the crystallinity of the samples A and B was about 33% and 31%, respectively.

### 3.2. Water Uptake and Conductivity

The results obtained from the analysis of the water uptake by the membranes equilibrated in liquid water at room temperature (Table 1) show that the sulfonated membranes we prepared based on deuterated sPS present similar hydration properties with those made of hydrogenated sPS with a comparable sulfonation degree, which were reported in [28].

The determination of the water uptake capacity is important for assessing the membrane functionality and performance in terms of the water and ion-transport, and the swelling and mechanical integrity. For this purpose, it is more suitable to express the amount of water that is taken up by the membrane as the number of water molecules per number of sulfonic acid sites, which defines the water content parameter, or the hydration number, *λ*≡n(H_2_O)/n(SO_3_H) [13]. The hydration number *λ* relates to the water uptake W_uptake_ (Equation (1)) using the equivalent weight of the membrane (EW, grams of dry polymer per ionic group), which is inversely proportional to the membrane ion-exchange capacity (IEC): *λ* = W_uptake_ × EW/M(H_2_O), with M(H_2_O)—the molecular weight of water (18 g mol^−1^). Assuming that the IEC of the s–sPS membranes is about 1.23 meq/g, similar to what we determined in [31] using the titration method, hydration numbers *λ* = 39.3 and 34.5 were obtained for our membranes A and B in fully hydrated state at room temperature, respectively. As a comparison, experiments provided values of *λ* in the range 20 to 30 for Nafion in the same hydration and temperature conditions [4]. Such high values of *λ* correspond to a bulk-like water regime that is reached as a consequence of the growth and connectivity of the hydrophilic domains when the membrane is equilibrated in liquid water. In this regime, the water molecules move freely, although still confined within nanodomains. The swelling of the hydrophilic domains as a consequence of water uptake is a multistep process [4], which starts with the formation of a hydration shell around the sulfonic acid groups and dissociation of protons, which become solvated and mobile at low hydration numbers (*λ* = 1 to 2)—the strongly bound water regime and continues with the formation of multiple solvation shells and water domains and the water percolation, in increasing the hydration level (*λ* up to 5 to 6). A further increase in hydration level leads to the growth and interconnecting hydrophilic domains and the transition from bound to free water (*λ* > 6). 

Usually the proton conductivity depends on the water uptake capacity of the membrane [4], which depends on its sulfonation degree [28]. The measured conductivity values of samples A and B at room temperature and at 80 °C are also reported in Table 1. At room temperature, the sample A, which has a higher sulfonation degree, is characterized by a higher conductivity than sample B. A detailed analysis of the dependence of proton conductivity on sulfonation degree in sPS-based membranes can be found in [24,26]. Both samples A and B show a higher proton conductivity at 80 °C compared to that exhibited at room temperature. As reported in the literature, the dependence of the proton conductivity on the temperature is Arrhenius-type [48,49], although deviations from this behavior can be observed at high temperatures and low hydration levels in some types of Nafion membranes [50,51]. However, at 80 °C, the sample B shows a much higher conductivity than the sample A, which seems to be an effect related to the incorporation of fullerenes into the membrane. An increase in conductivity of PEMs when fullerenes were added was observed in the case of Nafion 117. Conductivity of Nafion 117 and Nafion-C60 composite membrane as a function of RH and temperature is reported in [52]. The composite membranes performed better than the Nafion 117 over the temperature range from 20 °C to 80 °C and for different hydration levels between RH = 25% and 95%. Although the improvement in conductivity due to the addition of C60 (about 1 wt%, comparable to that in our sPS-based composite membranes) was moderate at high hydration level, under low humidity conditions, RH < 50%, the conductivity of the composite membranes was about three times higher than that of Nafion 117. On the other hand, the water uptake for the Nafion-C60 composite membranes shows only a little increase compared to Nafion 117 membranes. Although it is not clear why there is this tremendous increase in conductivity when fullerenes are added, possible morphological changes, which could not be observed at that low C60 loading, or the interfacial water between the C60 aggregates, which were revealed by the optical microscopy (OM) and scanning electron microscopy (SEM) observations, and the Nafion domains were suspected for this effect [52]. We can only speculate here that the same effects might be the reason for the observed much higher conductivity of the composite membrane at 80 °C compared to that of fullerenes-free membrane.

### 3.3. Microstructure Characterization

**Figure 3:**
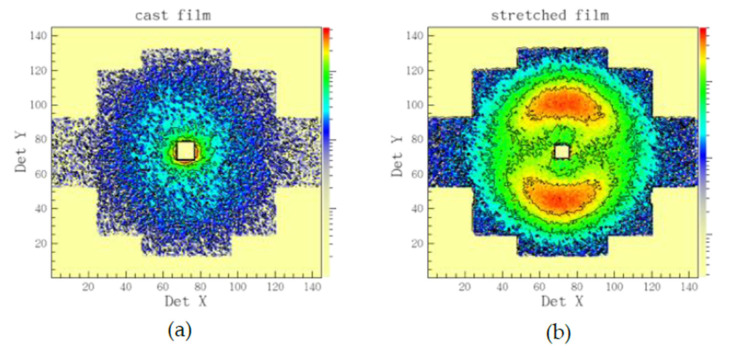
Example of typical two-dimensional neutron scattering patterns from sPS-based films as cast (**a**) or uni-axially deformed (**b**). The cast film delivers an isotropic scattering pattern on the two-dimensional position-sensitive neutron detector, while the stretched film, an anisotropic one, with the interlamellar correlation peaks from the oriented crystalline lamellar stacks appearing on meridian sectors (the stretching direction is vertical).

With SANS we investigated the evolution of the morphology of hydrated domains with variation of hydration level and temperature. As discussed in details in our previous publications [31,32], the cast films produce isotropic scattering patterns on the two-dimensional position sensitive detector, while uni-axially deformed films deliver on the detector clearly separated inter-lamellar peaks due to orientation of the lamellar stacks along the deformation axis. Thus, the two-dimensional scattering patterns from sample B are isotropic (Figure 3a), while those from sample A are anisotropic (Figure 3b). Therefore, on sample B a detailed qualitative analysis of scattering data at different RH and temperatures could be done, while a semi-quantitative analysis of data with structural models could be performed on sample A. The 010 crystalline peak should appear in the scattering patterns at very high Q if the sample preparation (stretching) and contrast would allow this [31]. In our study, the loading of deuterated polymer clathrates with d–toluene would however not enable this.

**Figure 4:**
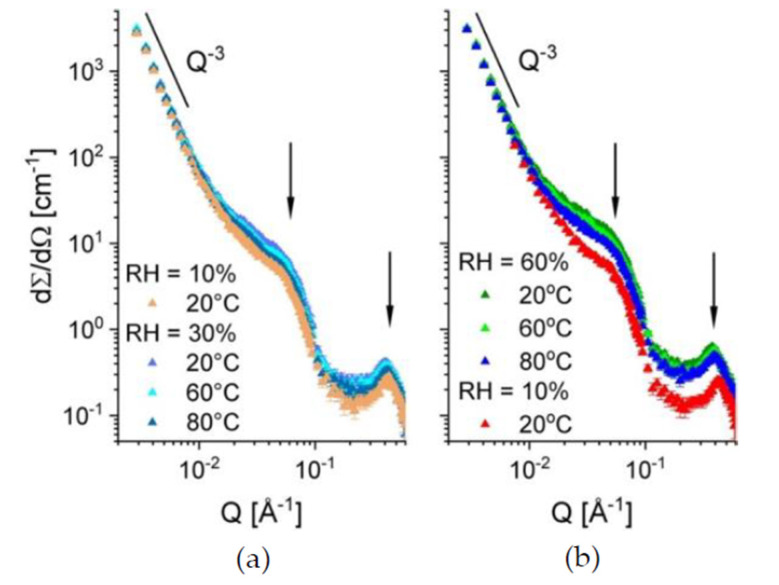
One-dimensional SANS patterns from sample B at different low (**a**) or intermediate (**b**) hydration levels and temperatures. The scattering features, which were observed in the scattering patterns and discussed in the text, are indicated by black arrows or the power-law scattering behavior.

In Figure 4, the one-dimensional scattering patterns from the sample B at different hydration levels and temperatures are displayed. The SANS results were collected for the sample treatment in the beam starting at RH = 10% and temperature of 20 °C (the orange curve in Figure 4a) and continuing at RH = 30%, varying the temperature between 20 °C and 80 °C (the three upper curves in Figure 4a). Further, the hydration was raised to RH = 60% and the temperature was varied again between 20 °C and 80 °C, followed by drying the sample back to RH = 10% and the temperature of 20 °C (Figure 4b). Three scattering features could be observed in all scattering patterns: a) the power-law behavior in the small Q-regime, where the typical upturn behaving like Q^−3^ is due to the large-scale fractal character of the polymer film; b) an intermediate Q-regime between 0.01–0.1 Å^−1^, where a broad feature corresponding to superposition of scattering signals from the inter-crystalline spacing (so-called “matrix knee”) and sulfonated domains appears; and c) the high Q-regime (around 0.1–0.5 Å^−1^), where the most characteristic feature is observed, namely the ionomer peak arising due to the correlation spacing between the dry or hydrated ionic clusters. After inspection of the evolution of scattering patterns and sample conditions, three main conclusions arise. (i) The scattering level in the intermediate Q-range, relevant for the length scale of the sulfonated and hydrated domains, increases with increasing the RH. This is due to absorption of more water by the film sample as the RH level increases, which leads to an increase in the neutron contrast between the hydrated (protonated) and the dry (deuterated) regions of the membrane. At the same time, the position of the ionomer peak shifts to lower Qs, as a consequence of the increase in the structural correlation length for the swelling ionic hydrophilic domains in the amorphous regions. (ii) The scattering level at constant RH decreases slightly with increasing the temperature. This may be due to a reduction in the absorbed water amount with increasing temperature that may be accompanied by morphological changes, hence changes in the contrast and size of the scattering objects (hydrated domains). (iii) The slight shift of the ionomer peak position towards higher Q values with increasing temperature at constant RH, accompanied by the slight decrease in intensity, as commented already in (ii).

This is an indication of a slight decrease in the correlation length between the ionic clusters with increasing temperature, which may be a consequence of weak morphological changes related to variation of the amount of the absorbed water. Also, morphological changes and changes in the micro-dynamics of the polymer matrix with increasing the temperature may induce such an effect. As reported earlier [32], the hydrated domain in sPS-based sulfonated membranes also includes segments of the sPS chain in addition to the sulfonic group attached to it. Therefore, it is worth studying in the future how the local micro-dynamics of the sPS at different temperatures may affect the conformation of the hydrated domains

**Figure 5:**
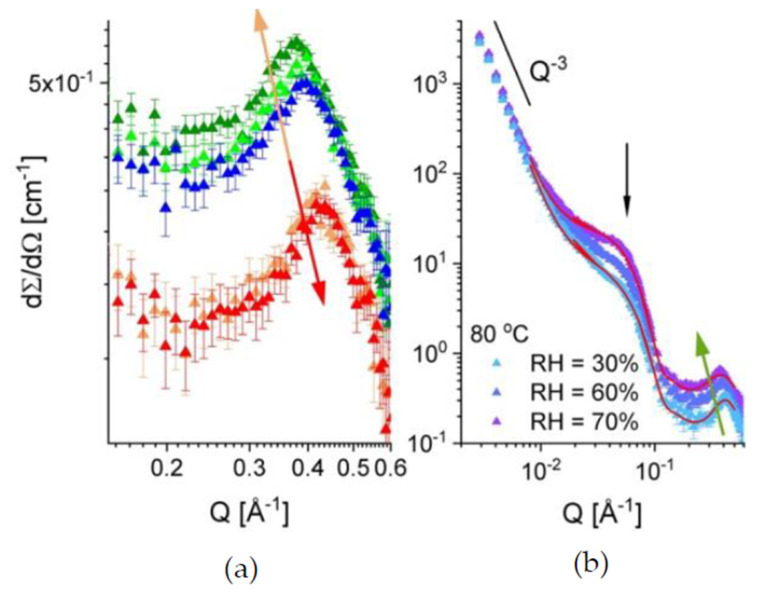
The evolution of the ionomer peak in sample B (indicated by arrows) with increasing the RH and temperature followed by the return to the initial hydration and temperature state (**a**), and the one-dimensional scattering patterns from the same sample at 80 °C with varying the RH (**b**). The colors in Figure 5a are as in Figure 4. The red curves in the Figure 5b represent the model description of the experimental curves (see text) while the green arrow indicates the evolution of the ionomer peak with increasing the RH.

After applying variation of RH and temperature on the membrane, the scattering pattern from the film sample B exposed again to RH = 10% and the temperature of 20 °C (red curve in Figure 4b) coincides with that in the initial state of the membrane. This can be very well observed in Figure 5a, where the evolution of the ionomer peak profile as the RH and temperature are varied and then set back to initial values of the sample treatment in beam. As expected, the peak position moves to lower *Q* values (orange arrow) when the RH is raised from 10% to 60%, while having the same temperature on the sample (20 °C), which is indicative for the swelling of the hydrated domains and the increase in the correlation length between the ionic clusters.

At RH = 60%, the peak position moves slightly to higher Q values as the temperature is increased. A slight shrinkage of the hydrated domain and a decrease in the correlation length between the ionic clusters seem to take place. Changing back the RH to 10% and the temperature to 20 °C makes the ionomer peak recover its initial profile (red arrow). Figure 5b, presents the scattering profile from the same membrane at 80 °C for different hydration levels. The evolution of the scattering features with increasing RH conforms to the discussion made above at the point (i). As mentioned in the beginning of this paragraph, the scattering at intermediate Q from cast films, which consist of functionalized semi-crystalline polymers such as sPS, arises as a superposition of scattering signals from the dry/hydrated amorphous domains and the crystalline regions (the inter-lamellar correlation). However, due to the random orientation of the crystalline lamellae in a cast film, the later signal is smeared out and has a weak contribution to the scattering at intermediate Q, which occurs mainly from the functionalized amorphous domains. When these domains are hydrated, their scattering contribution prevails at intermediate Q. Therefore, we tried to get a first insight on the morphology and volume fraction occupied by the hydrated domains (assumed as scattering particles) within the sample by interpreting the data in terms of the simple model of scattering from correlated spherical objects:

(3) $$I\left(Q\right)\;=\;\varphi ∆\rho^{2}V_{d}P\left(Q\right)S\left(Q\right){\;+\;I}_{\mathrm{ion}}{\;+\;I}_{\mathrm{fract}}\;+\;\mathrm{Bckgd}$$

where P(Q) represents the particles form factor, which relates to the intra-particle correlations, and S(Q) the structure factor, which denotes the inter-particle correlation effects, and are described in details in [32]. The contrast Δρ = ρ_d_ − ρ_env_ is the difference between the SLD of the scattering hydrated domains ρ_d_ and their polymeric environment. Usually, the factor I_0_ = (φ Δρ^2^ V_d_) is called the “forward scattering” from the ensemble of scattering objects. The terms I_ion_ and I_fract_ represent the additional contribution at high Q, from the ionomer peak, which can be described by a Gaussian function, and at low Q, from the fractal behavior of the film, which can be described by a simple power-law term, P_3_Q^−3^, with P_3_ the power-law constant [32]. A constant background, Bckgd, which arises mostly from the incoherent scattering contribution from the film sample, is added as a final term of the model. The red lines in Figure 5b, represent the model interpretation of the experimental data collected for RH = 30% and 70%. The main parameters of the fitting procedure were the radius of the hydrated domains R_sph_, assumed spherical, and the “forward scattering” from these domains, (I_0_)_sph_. Other free parameters were considered in the fitting procedure: the P_3_; the area, width and position of the Gaussian that describes the ionomer peak; and the parameters defining the structure factor in Equation (3) [31]. Despite the multitude of free parameters, the reliability of the fitting procedure is high, because the three structures considered—the fractal behavior, the hydrated domains and the correlation between the ionic clusters, are very well separated by sizes and their contributions to the total scattering curve overlap only marginally over the wide Q-range covered in this experiment. This makes the parameters that describe them pretty well determined. Moreover, at RH = 30% it seems that the spherical water domains are still well separated from each other: The spherical form factor is well defined towards high Qs and no shoulder or peak-like feature is observed, as in the case of the data measured at RH = 70%, when the S(Q) contribution yields the additional scattering observable as a shoulder at around Q = 0.07 Å^−1^ (indicated by the vertical arrow). The dimensions of the hydrated domains formed at RH = 30% and 70% are about R_sph_ = 48 Å and 54 Å, respectively, quite smaller to the values reported for the fullerene doped s–sPS membranes [32].

However, the C60–sPS composite membrane (sample B) investigated in this study was produced following a different procedure than the previously studied membranes. On the other hand, the volume fraction occupied by the hydrated domains in the amorphous region of the film sample B, which was estimated from the interpretation of the (I_0_)_sph_ by taking into account the crystallinity of the film, is about 2.35% and 8.52% at RH = 30% and 70%, respectively. The value obtained at RH = 70% seems to be larger than that obtained for the fullerene doped sPS membranes [32], which were characterized by a slightly higher sulfonation degree than the film sample B in this study. Although the values are not very different from each other, we should take into consideration that the current values were obtained for the sample heated at 80 °C, where, according to our qualitative SANS observations, the amount of the adsorbed water should be lower than that at room temperature. Apparently, the presence of fullerenes in the composite membrane and the tendency of fullerene to aggregate seem to have an effect on the morphology and content of the water domains in the composite membrane.

**Figure 6:**
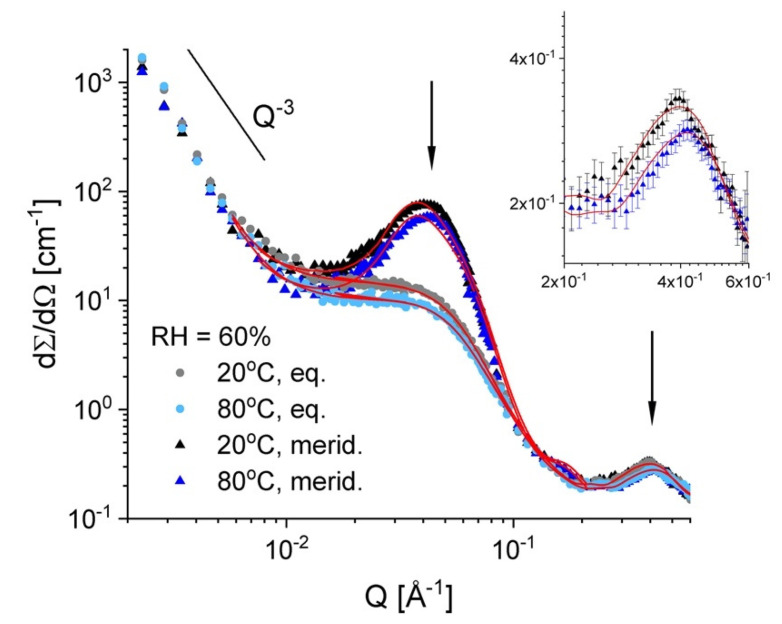
One-dimensional SANS patterns from the film sample A at RH = 60% and different temperatures. Experimental data (symbols) are averaged over the equatorial and meridian directions, with the lines corresponding to the model interpretation of the scattering profiles, as discussed in text. The main scattering features are indicated by the power-law and the vertical arrows. The inset presents in details the ionomer peak (the patterns over the meridian sectors, as long as this scattering feature appears isotropic in the two-dimensional scattering pattern).

Figure 6 presents the scattering data from sample A at RH = 60% and two temperatures, 20 °C and 80 °C, as they were averaged over the equatorial and meridian sectors of the anisotropic two-dimensional scattering patterns that form the uni-axially deformed membrane (Figure 3b). The slight decrease of the scattering level with increasing the temperature is observed here too, as it was in the case of sample B. The scattering features from different microstructural levels occurring in the membrane as a consequence of its properties and treatment—the semi-crystalline character, the functionalization (sulfonation) and the hydration, are clearly revealed by the scattering patterns on different detection sectors due to alignment of some of these structures under stretching [31]. The low-Q power law behavior due to the fractal character of the membrane at a larger length scale, around 1000 Å, is visible in the scattering profiles collected on both the equatorial and meridian sectors. The ionomer peak, which is an isotropic scattering feature, is also visible in the high-Q data on both equatorial and meridian sectors. At intermediate Q, the isotropic scattering from the hydrated domains is well distinguished in the data on equatorial sectors, while the same feature on the meridian sectors is buried under the very strong scattering from the oriented lamellar stacks. Sample A was measured with the stretching direction positioned vertically in the neutron beam, therefore, the strong reflections due to the inter-lamellar correlation appear in the meridian direction (Figure 3b). The scattering data on meridian and equatorial sectors were fitted simultaneously for each temperature according to the model that is presented in detail in [32]. From the interpretation of the fitted parameters, the main geometrical and density information about the hydrated domains in the amorphous regions could be obtained in a similar way as described in [32]. The fitting procedure delivered the size of the hydrated domains and their “forward scattering”. Supposing spherical hydrated domains, the volume fraction occupied by water in the amorphous region could be estimated from the evaluation of the forward scattering. The fitting procedure also delivered the SLD of the hydrated inter-lamellar amorphous regions, from which the volume fraction occupied by water in these regions could be estimated. The evaluated parameters are reported in Table 2. As it can be observed, the volume fraction in the membrane that is occupied by the water is lower at 80 °C than at 20 °C, which agrees with the observation made on sample B. The volume fractions occupied by water within the bulk and inter-lamellar amorphous regions are comparable at 20 °C and are quite similar to those evaluated for the fullerenes-doped sPS membranes for comparable sulfonation degree, which were reported in [32].

On the other hand, at 80 °C it seems that the volume fraction of water within the inter-lamellar amorphous regions is slightly higher than that in the bulk amorphous domains. Apparently, at high temperature, the water desorbs easier from the bulk amorphous than from the interlamellar amorphous regions. The average size of the hydrated domains is also slightly smaller at 80 °C than at 20 °C (Table 2). The correlation length between the hydrated ionic clusters, ξ_ion_ = 2π/Q_ion_, where Q_ion_ is the ionomer peak position, shows the same trend (Table 2).

**Figure 7:**
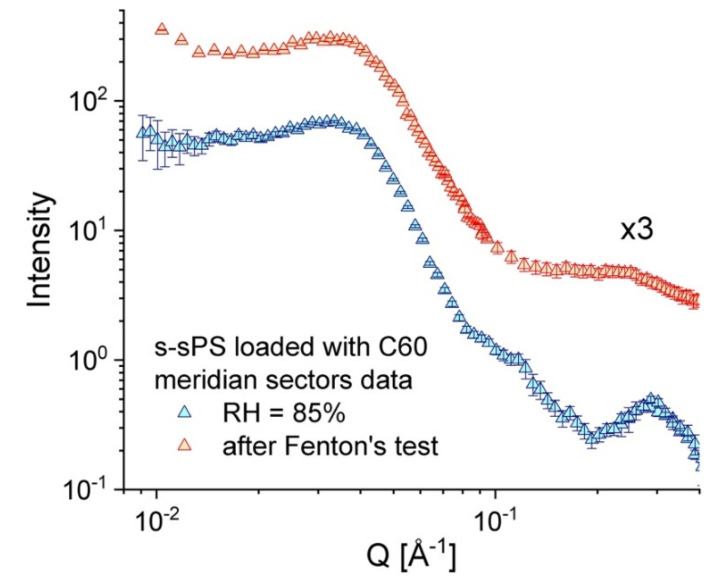
One-dimensional SANS patterns over the stretching direction from a uni-axially deformed s–sPS film incorporating C60 fullerenes, as measured in different treatment conditions: blue symbols—the sample at RH = 85% and room temperature (data reported [32]); red symbols (vertically shifted for clarity)—the sample exposed to the Fenton’s test conditions.

Finally, the brief exposure of an old uni-axially deformed s–sPS film (sulfonation degree S = 19.5%, crystallinity of 22%, and doped with C60 fullerenes [32]) to the Fenton’s test conditions [27] seems to have no effect on the crystalline phase of the membrane, as can be deduced from the inspection of the scattering patterns from the tested film, compared to those from the same sample before the application of the test. Figure 7 shows the scattering data averaged over the sectors parallel with the deformation axis of the membrane after it was kept for 1 h in Fenton’s reagent at 60 °C (red symbols) in parallel with the SANS data from the same membrane at RH = 85% (blue symbols, data already discussed in [32]), before the Fenton’s test was carried out. In both cases the inter-lamellar peak due to correlation effects between oriented lamellae in the crystalline phase of stretched film can be well observed at around Q * = 0.035 Å^−1^, which is indicative of the preservation of membrane crystalline features regardless of its treatment. The profile of the ionomer peak at high Q values, however, is different, depending on the membrane treatment: it is clearly observed in the scattering pattern from the hydrated membrane at RH = 85%, while after immersing the polymer film into the Fenton’s reagents, the peak turns into a very broad feature and shifts to lower Q values due to the extreme hydration of the membrane and the high incoherent scattering contribution of the absorbed water.

This test offers a first hint about the resistance to oxidative degradation of the sulfonated membranes containing fullerenes that we have prepared based on sPS, and confirms the early observations made on similar systems, as reported in [27]. However, further systematic tests of film samples with different fullerene contents must be carried out over a longer time in different treatment conditions (higher temperature and involvement of higher concentration reagents), in order to clearly assess the oxidative stability and life time of the membranes of this type. On the other hand, it seems that the addition of fullerene does not significantly improve the mechanical properties of sPS-based membranes [27], which apparently are still poorer than those shown by Nafion, though thorough mechanical examination still needs to be performed for quantitative conclusion on this issue.

## 4. Conclusions

The structural results of this study enabled a characterization of the hydrated domains in the sPS-based membranes in different humidity and temperature conditions. Our complex structural study presented here completes the conclusions reported in the previous publications [31,32]. According to these, the water that is taken-up by the functionalized membranes accumulates around the agglomerations of sulfonic groups and gives rise to hydrated domains, mostly in the bulk amorphous region. The hydrated domains grow in size and number with increasing the hydration level while, on the other hand, they shrink slightly with increasing the temperature at constant hydration level, due to desorption of some water, mostly from the bulk amorphous regions. Despite these weak morphological changes at high temperatures, the conductivity of the membrane seems not to be affected, as observed in [30]. With a further increase of hydration level up to the complete membrane equilibration in liquid water, the water clusters grow and give rise to water channels.

According to our measurements, the sulfonated sPS–fullerenes composite membranes perform at high temperature much better than the fullerenes free membranes in terms of proton conductivity in liquid water. Apparently, this may be related to the formation of additional hydrated pathways in the composite membrane due to the interfacial water accumulated between the fullerene aggregates and the sulfonated polymer domains, like in the case of Nafion-fullerenes composite membranes [52], possibly in conjunction with changes in the dynamics of water and polymer at high temperatures. To better understand this observed effect in the sPS-based membranes and to verify these assumptions, further investigations of the micro-structure and micro-dynamics in such systems are needed, possibly involving also a much simpler system, such as the amorphous atactic polystyrene.

## Figures and Tables

**Table 1:** The characteristics of the sPS-based films after the sulfonation and hydration procedures.

Parameter	Sample A	Sample B
Sulfonation degree (%)	46.3	41.5
Crystallinity (%)	33	31
Water uptake at 25 °C (%)	87.5	76.3
Conductivity 25 °C (mS/cm)	128.2	99.9
Conductivity 80 °C (mS/cm)	160.3	219.8

**Table 2:** The structural parameters of the hydrated morphologies in samples A and B of this study, as delivered by the interpretation of the experimental data according to the models introduced in [32].

Parameter	Sample A, RH = 60%		Sample B, 80 °C	
	20 °C	80 °C	RH = 30%	RH = 70%
(φ_water_)^amorphous^ (%)	3.24	2.75	2.35	8.52
R_sph_ (Å)	50.3	45.1	41.5	38.2
(φ_water_)^inter-lam^ (%)	3.21	2.87	–	–
ξ_ion_ (Å)	16.10	14.95	16.75	15.06
